# The FAT1 Cadherin Drives Vascular Smooth Muscle Cell Migration

**DOI:** 10.3390/cells12121621

**Published:** 2023-06-14

**Authors:** Dario F. Riascos-Bernal, Gaia Ressa, Anish Korrapati, Nicholas E. S. Sibinga

**Affiliations:** 1 Department of Medicine (Cardiology) and Wilf Family Cardiovascular Research Institute, Albert Einstein College of Medicine, Bronx, NY 10461, USA; gaiaressa@gmail.com (G.R.); anish.korrapati@gmail.com (A.K.); 2 Department of Developmental and Molecular Biology, Albert Einstein College of Medicine, Bronx, NY 10461, USA

**Keywords:** FAT1, cadherin, cell migration, vascular smooth muscle cell, vascular disease, Atrophin, RERE, angiotensin II

## Abstract

Vascular smooth muscle cells (VSMCs) are normally quiescent and non-migratory, regulating the contraction and relaxation of blood vessels to control the vascular tone. In response to arterial injury, these cells become active; they proliferate, secrete matrix proteins, and migrate, and thereby contribute importantly to the progression of several cardiovascular diseases. VSMC migration specifically supports atherosclerosis, restenosis after catheter-based intervention, transplant vasculopathy, and vascular remodeling during the formation of aneurysms. The atypical cadherin FAT1 is expressed robustly in activated VSMCs and promotes their migration. A positive role of FAT1 in the migration of other cell types, including neurons, fibroblasts, podocytes, and astrocyte progenitors, has also been described. In cancer biology, however, the effect of FAT1 on migration depends on the cancer type or context, as FAT1 either suppresses or enhances cancer cell migration and invasion. With this review, we describe what is known about FAT1’s effects on cell migration as well as the factors that influence FAT1-dependent migration. In VSMCs, these factors include angiotensin II, which activates FAT1 expression and cell migration, and proteins of the Atrophin family: Atrophin-1 and the short isoform of Atrophin-2, which promote VSMC migration, and the long isoform of Atrophin-2, which exerts negative effects on FAT1-dependent VSMC migration.

## 1. Introduction

Quiescent, non-migratory, contractile vascular smooth muscle cells (VSMCs) are the main cellular component of the medial layer of arteries and play a critical role in controlling the tone and diameter of the vessel. In response to vascular injury, however, VSMCs lose their expression of contractile proteins and acquire the ability to proliferate and migrate from the media to the subendothelial space, contributing to both medial and intimal thickening [1]. This VSMC phenotypic transition (or switching) is associated with the progression of several cardiovascular diseases including atherosclerosis, restenosis after angioplasty and stent placement, graft vascular disease, and hypertension.

VSMC migration contributes to fibroatheroma formation in atherosclerosis, to the remodeling of the arterial wall in aneurysm formation, and to neointima formation in in-stent restenosis and transplant vasculopathy [2,3,4,5]. Given the pathogenic role of VSMC migration in cardiovascular disease, better understanding of the molecular mechanisms underlying this cellular activity may be instrumental for the development of strategies that modulate VSMC migration and mitigate disease burden.

In general, distinct environmental signals including chemotactic, haptotactic, and durotactic cues stimulate VSMC migration by activating signal transduction mechanisms that, in turn, induce the remodeling of both the cytoskeleton and cell adhesions. Directional migration is a cyclic process that entails: (a) Activation by extracellular signals of cell surface receptors, typically receptor tyrosine kinases or G-protein-coupled receptors; (b) Induction of cell polarity and filopodia, followed by lamellipodia formation and protrusion of the leading edge; (c) Attachment of the leading edge by the formation of new cell-extracellular matrix adhesions; (d) Actin- and myosin-mediated contraction that pulls the cell body forward; (e) Disassembly of cell–extracellular adhesions at the trailing edge, which, in association with the actin- and myosin-dependent contraction, results in detachment of the rear end and movement of the cell forward [6,7,8]. The molecular factors involved in each of these steps have not been fully elucidated.

Activated VSMCs express higher levels of FAT1 compared to non-migratory VSMCs; indeed, known chemotactic factors such as platelet-derived growth factor (PDGF) induce FAT1 expression [9]. FAT1, an atypical cadherin, is a large transmembrane protein that is localized to membrane protrusions such as filopodia and lamellipodia and promotes VSMC migration; interestingly, FAT1 undergoes processing, and its intracellular domain (FAT1ICD) can also be found in the nucleus or in mitochondria [9,10,11]. Notably, mouse or rat arteries undergoing remodeling after vascular injury as well as sections of human atherosclerotic arteries adjacent to sites of stent placement show abundant FAT1 expression that follows the expression pattern of VSMC markers [9,11]. Nevertheless, our current understanding of atypical cadherins (including FAT1) in VSMC biology is incipient. Interestingly, other structurally distinctive atypical cadherins such as CELSR and cadherin-13 (T-cadherin) have also been linked to effects on cell migration [12,13].

We performed a Pubmed search to identify papers linking FAT1 to effects on cell migration, with an eye to understanding how this protein contributes to VSMC migration. Interestingly, our search identified 55 primary reports and 8 reviews linking FAT1 to migration in different cell types. In this review, we report on studies that associate the FAT1 cadherin with cell mobility, including both pro- and anti-migratory effects. To provide a more comprehensive picture of the molecular mechanisms underlying these effects, the studies we discuss are not limited to VSMCs; specifically, we include reports of FAT1 in cancer cells in which anti-migratory function has been described. In brief, the pro-migratory mechanisms identified suggest that FAT1 interacts with proteins involved in actin dynamics and cell migration; anti-migratory FAT1 effects may be related at least in part to changes in the cell state since FAT1 opposes the epithelial–mesenchymal transition (EMT) [14,15] and associated increases in cell migratory ability. In addition, angiotensin II-dependent activation of NADPH oxidase 1 and the long isoform of Atrophin-2 may serve as positive and negative upstream regulators of FAT1-dependent VSMC migration, respectively.

## 2. Key Points

### 2.1. The FAT1 Cadherin Is a Transmembrane Protein with a Distinct Cellular Distribution

**Figure 1:**
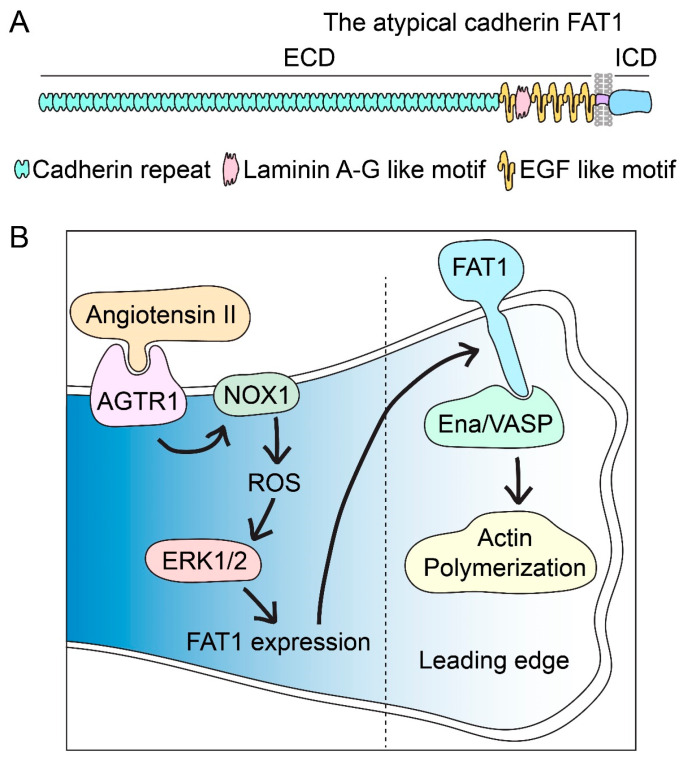
FAT1 cadherin promotes vascular smooth muscle cell migration. (**A**) The structure of the atypical FAT1 cadherin includes a large extracellular domain (ECD), a single-spanning transmembrane domain, and an intracellular domain (ICD) with homology limited to that of classical cadherins. The ECD contains thirty-four cadherin repeats, five epidermal growth factor (EGF)-like motifs, and one laminin A-G domain. (**B**) FAT1 expression is induced in migratory VSMCs by chemotactic factors such as angiotensin II. Angiotensin II activates angiotensin II receptor 1 (AGTR1), which in turn stimulates NADPH oxidase 1 (NOX1) and reactive oxygen species (ROS) generation. Subsequently, ROS-dependent activation of extracellular signal-regulated kinase (ERK)1/2 may increase FAT1 expression. FAT1 is localized to the leading edge, wherein it interacts through its intracellular domain with proteins of the Ena/VASP family such as mammalian-enabled (Mena, also known as ENAH actin regulator) or vasodilator-stimulated phosphoproteins (VASPs). Ena/VASP proteins connect FAT1 with actin polymerization-favoring membrane protrusion formation and migration.

The FAT1 cadherin, a member of the cadherin superfamily, is the protein product of the *FAT1* gene located at chromosome 4q35.2. The *FAT1* transcript, initially cloned in 1995 from a T-cell leukemia line, was predicted to encode a large type I transmembrane protein of 4588 amino acids containing thirty-four cadherin repeats, five epidermal growth factor (EGF)-like repeats, and a laminin A-G domain in its extracellular domain; in addition, FAT1 was also predicted to have a single-spanning transmembrane domain and an intracellular domain bearing homology, albeit limited, to that of classical cadherins (Figure 1A) [16]. Although *FAT1* was initially considered to be the homolog of Drosophila *fat* [16], a later study argued that *FAT1* is an ortholog of the Drosophila *fat-like* gene, which is involved in the normal development and maintenance of tubular structures such as the trachea, proventriculus, salivary glands, and hindgut [17].

FAT1 expression is abundant in different tissues during embryonic development but scarce in normal adult tissues [16,18]. Although a thorough evaluation of FAT1 expression in the vasculature during development is lacking, FAT1 is expressed in arterial SMCs in the human fetal eye and in the rat aortic outflow tract during development [16,19]. The first description of FAT1 expression in the vasculature in adulthood showed that normal rat arteries exhibit very low levels of *Fat1* mRNA or protein, whereas arteries undergoing remodeling due to balloon injury show abundant FAT1 expression in the media and neointima [9]; subsequent studies showed a similar pattern of expression in normal or ligated carotid mouse arteries [11]. Notably, cross-sections of human coronary arteries affected by atherosclerosis and adjacent to previous sites of stent placement exhibit clear FAT1 protein expression that follows the pattern of VSMC markers [11].

Consistent with its transmembrane protein structure, FAT1 in VSMCs is localized to the plasma membrane, for instance, in lamellipodia and filopodia at the leading edge and at regions of cell–cell contact [9]. Notably, the nucleus and mitochondria of VSMCs also exhibit a specific signal for FAT1 in immunofluorescence studies using an antibody against the intracellular domain by the C-terminus of the protein [9,11]; in addition, cell fractionation studies also confirm the presence of FAT1 intracellular domain species in mitochondria [11]. While FAT1 fragments within mitochondria interact with respiratory complexes, limit respiratory supercomplex formation, and act to restrain VSMC mitochondrial respiration [11], the functional consequences of FAT1 in the nucleus remain to be elucidated.

Similarly, the proteolytic cleavage processes that generate free intracellular domain species that translocate to the nucleus or mitochondria and the process of mitochondrial or nuclear transport are not yet understood in VSMCs. Interestingly, the Drosophila fat cadherin undergoes proteolytic cleavage to release an intracellular domain fragment that translocates to the mitochondria, wherein it stabilizes respiratory complex I [20]. These observations suggest a schema conserved from Drosophila to mammals by which atypical cadherins expressed in the membrane may sense extracellular signals and relay this information to mitochondria, providing a relatively direct mechanism to control cellular respiration.

### 2.2. The FAT1 Cadherin Supports the Migration of Cells Other than VSMCs

To understand how FAT1 affects VSMC migration, information from studies of FAT1 in the migration of other cell types may be instructive. Here, we discuss findings in muscle progenitors, fibroblasts, astrocytes, neurons, and renal epithelial cells that help to portray the complexities surrounding FAT1 in cell migration.

During neuromuscular morphogenesis, the coordinated polarity and collective migration of muscle progenitors are required for the development of skeletal muscles with a functional shape [21]. In this setting, muscle progenitors, mesenchymal cells, and subsets of motor neurons express FAT1 [22]. Interestingly, the conditional genetic deletion of FAT1 in mice indicates that FAT1 is required for the migration of muscle progenitors in a cell-autonomous manner [21]. In addition, the loss of FAT1 in either motor neurons or in mesenchymal cells also impairs muscle progenitor migration in an indirect manner [22]. Thus, FAT1 is an important factor for skeletal muscle formation, fulfilling complementary roles in different cell types, and the loss of FAT1 function seems to be relevant to pathologies of the skeletal muscle such as facioscapulohumeral dystrophy [21,22].

In human fibroblasts, homozygosity in a loss-of-function FAT1 mutation impairs migration, which is partially improved by the activation of RAC1 and CDC42, members of the Rho family of GTPases known to regulate many aspects of actin dynamics [23]. In the same study, FAT1 loss in differentiated podocytes in culture inhibited cell migration, and this was also partially rescued by RAC1 and CDC42 activation. Moreover, FAT1 knockdown impaired migration in a renal tubular cell line. These observations suggest that FAT1 promotes the cell migration of glomerular and renal tubular cells in part by activating RAC1 and CDC42; perturbation of this mechanism seems to be relevant to the pathogenesis of steroid-resistant nephrotic syndrome [23].

In the postnatal development of the retina, the astrocyte layer guides endothelial cells participating in retinal angiogenesis [24]. During this process, FAT1 expression is predominant in astrocytes and Mϋller glia [24]. Genetic studies in mice show that FAT1 expression in migrating astrocyte progenitors is required for their proper migration polarity at the leading edge; moreover, FAT1 is also necessary for the migration of endothelial cells in a non-cell-autonomous manner, as the neuronal-specific loss of FAT1 affecting astrocytes also delays retinal angiogenesis [24].

In epithelial and neuronal cells, FAT1 expression is localized to leading-edge lamellipodia and filopodia and to intercellular junctions, as well as along the shaft of retraction fibers [25,26]. Notably, FAT1 knockdown in epithelial cells impaired migration in a scratch-wound assay, and this was associated with deficient lamellipodia formation and the loss of cell polarity [25]. Interestingly, in the process of neural tube closure during mouse development, FAT1 knockdown seems to impair neuronal migration [27].

Mechanistically, the FAT1 intracellular domain interacts with vasodilator-stimulated phosphoproteins (VASP) and Mena (the product of the *ENAH* gene), both of which belong to the Ena/VASP family of proteins known to be involved in actin dynamics; this interaction may explain in part the requirement of FAT1 for lamellipodia formation but does not necessarily account for the requirement of FAT1 for cell polarity [25,26].

Beyond the proteolytic processing already described, the additional complexity of FAT1 biology stems from the presence of multiple isoforms generated by differential RNA splicing, particularly those affecting the composition of the FAT1 intracellular domain. Following the nomenclature proposed by Braun et al. [28], studies show that in addition to wild type FAT1, several mouse tissues including those of the kidney, liver, lung, and ileum express a FAT1 isoform, designated FAT1(+12), that contains 12 additional amino acids in the intracellular domain [28]. The brain, in contrast, expresses wild type FAT1; FAT1(+32), which has 32 additional amino acids in the intracellular domain; and FAT1(+8TR), which is characterized by a premature stop codon and a truncated FAT1 intracellular domain [28]. The dominant isoform expressed in VSMCs is FAT1(+12), but the vasculature has not been fully evaluated for FAT1 isoform expression. In a renal epithelial cell line, FAT1(+12) expression is localized exclusively at sites of cell–cell contact and is absent from leading-edge lamellipodia or filopodia; these membrane protrusions, which are important for cell migration, appear to contain only wild type FAT1 [28]. Moreover, non-migratory cells have higher levels of FAT1(+12) and lower levels of wild type FAT1 compared to migratory cells; importantly, the specific knockdown of the FAT1(+12) isoform increases the number of cell protrusions per cell and cell migration in a wound healing assay, whereas cells deficient in wild type FAT1 exhibit impaired cell migration [28]. Interestingly, an analysis of human kidney biopsies showed that normal glomeruli predominantly express wild type FAT1 over FAT1(+12), whereas minimal change disease, focal segmental glomerulosclerosis, and membranous glomerulonephritis exhibit the inverse pattern of expression [28].

In summary, FAT1 cell-autonomously promotes migration in several cell types, including skeletal muscle and astrocyte progenitors, fibroblasts, some epithelial cells, and podocytes and renal tubular cells [23,24,25,26]. In addition, via non-cell-autonomous mechanisms, FAT1 supports the migration of endothelial cells during retinal angiogenesis [24] and the migration of skeletal muscle progenitors [21,22]. Interactions of the FAT1 intracellular domain with Ena/VASP proteins potentially link the former to dynamic actin regulation. Different splice isoforms affecting the composition of the FAT1 intracellular domain have been linked variably to cell migration vs. cell–cell contact; the relation of these isoforms to Ena/VASP interactions and autonomous vs. non-autonomous mechanisms is not known.

### 2.3. The FAT1 Cadherin May Promote or Oppose Cancer Cell Migration

The studies of non-neoplastic cells described above depict FAT1 as either pro-migratory or neutral in its effects on cell migration. In general terms, FAT1 has been reported to oppose the epithelial–mesenchymal transition (EMT) [14]; this suggests that the loss of FAT1 would promote a mesenchymal, and therefore more migratory, phenotype. While this is true for some cancers, other reports describe FAT1 as pro-migratory. Here, we briefly summarize studies that report an effect of FAT1—negative or positive—on cancer cell migration, as understanding its disparate effects in different cancer types could lead to a greater understanding of how FAT1 affects VSMC migration.

The following studies support the Idea that FAT1 promotes cancer cell migration and invasion. Cell lines of grade IV glioblastoma multiforme exhibit higher FAT1 expression than grade III glioma cell lines; in addition, FAT1 knockdown in grade IV cell lines reduces cell migration and invasion and increases the expression of programmed cell death 4, a known tumor-suppressor; moreover, the migration and invasion of glioma cells are restored in FAT1-deficient cells by the simultaneous knockdown of programmed cell death 4 [29]. FAT1 expression is elevated in human samples of gastric cancer as well as in a model of this disease, and the knockdown of FAT1 in gastric cancer cell lines impairs cell migration and invasion [30]. Furthermore, FAT1 knockdown inhibits, while FAT1 overexpression enhances, cell migration and invasion in gastric or bladder cancer cell lines [31,32]. On the other hand, a circular RNA species derived from the FAT1 locus, circFAT1, is elevated in colorectal cancer tissues and cells as well as in papillary thyroid cancer cell lines, and circFAT1 knockdown impairs migration and invasion in both colorectal and papillary thyroid cancer cell lines [33,34]. Similarly, circFAT1 generated from exon 2 is upregulated in osteosarcoma tissues and cell lines, and the knockdown of circFAT1 prevents the migration and invasion of osteosarcoma cells [35]. How the effects of circFAT1 at a molecular level relate to other FAT1 effects on migration is not yet clear.

On the other hand, a number of studies report that FAT1 suppresses cancer cell migration and invasion, which is more consistent with the functions of a classical cadherin. FAT1 expression is lower in both esophageal squamous cell carcinoma and cervical cancer; notably, FAT1 knockdown promotes migration and invasion in esophageal squamous cell carcinoma cell lines and of HeLa and C33A cells, while FAT1 overexpression abrogates these cellular activities [14,36,37]. In addition, in esophageal squamous cell carcinoma cell lines, the knockdown of circFAT1 increases migration and invasion [38]. With regard to lung cancer, FAT1 expression was found to be upregulated in a non-small-cell lung cancer cell line that exhibited decreased migration and invasion [39]. On the other hand, in head and neck squamous cell carcinoma, FAT1 loss-of-function mutations are associated with decreased FAT1 expression in tumors; interestingly, FAT1 knockdown increases migration and invasion in head and neck squamous cell carcinoma cell lines [40].

Still, other studies suggest that FAT1 may induce or inhibit cancer cell migration and invasion of the same tumor type in a context-dependent manner. FAT1 expression is elevated in both hepatocellular carcinoma and oral squamous cell carcinoma cell lines; notably, FAT1 knockdown impairs the migration or invasion of these cells [41,42,43,44,45]. On the contrary, when cells were grown in a simulated blood environment, FAT1 knockdown in hepatocellular carcinoma cell lines enhanced cell migration and invasion [46], and the loss of FAT1 in oral squamous cell carcinoma cells increased cell migration in a different study [47].

Whether these differences in how FAT1 affects cancer cell migration stem from variable effects on EMT or the differential expression of various FAT1 isoforms with distinct subcellular localizations and/or functions, as noted above, is not known. Alternatively, differences in Rac1/CDC42 levels or regulation, or in Ena/VASP protein expression might affect how particular cells are able to migrate. Such determinations will require further testing in different cancers, but these same variables could affect how FAT1 affects VSMC migration.

### 2.4. Full-Length FAT1 Cadherin Supports Vascular Smooth Muscle Cell Migration

The FAT1 cadherin is highly expressed in the arterial wall in settings characterized by enhanced VSMC migration including the response to vascular injury and disease. FAT1 expression is induced at the mRNA and protein levels in rat carotid arteries upon balloon injury; this phenomenon is initially observed in the medial layer of the artery and subsequently becomes predominant in the developing neointima [9]. FAT1 expression is also upregulated in a similar pattern in mouse carotid arteries after vascular ligation and, remarkably, in regions of human atherosclerotic coronary arteries adjacent to sites of stent placement [11]. In addition, FAT1 expression increases specifically in primary cultured VSMCs in response to factors known to stimulate VSMC migration. In primary cultured rat aortic VSMCs, FAT1 expression is induced by fetal bovine serum as well as by angiotensin II, basic fibroblast growth factor (FGF), and PDGF-BB, which are known chemotactic factors [9]. This pattern of expression in vitro and in vivo suggests that FAT1 may play a role in VSMC migration.

Loss-of-function studies of FAT1 in VSMCs support the idea that full-length FAT1 promotes cell migration in that the reduced expression of full-length FAT1 proteins achieved by siRNA-mediated knockdown decreases the migration of primary VSMCs in culture. Surprisingly, overexpression of the FAT1 intracellular domain using an engineered construct—in which the extracellular and transmembrane domains of the interleukin 2 receptor α-chain are fused to the FAT1 cytoplasmic region—also inhibits this cellular activity [9]. Together, these findings suggest that full-length FAT1 is necessary for VSMC migration and that the FAT1 extracellular domain (and possibly the transmembrane region) is essential in this function. The intracellular domain, on the other hand, is not sufficient to promote this activity and, in fact, may oppose VSMC migration, at least when separated from the other FAT1 domains [9]. Such an effect could reflect dominant negative interference with normal FAT1 interactions; candidate interactors for such mechanisms include the Ena/VASP proteins, which associate with FAT1 intracellular sequences in epithelial cells [25,26] and in VSMCs [9]. Ena/VASP proteins are involved in actin polymerization and thus may connect full-length FAT1 with VSMC cytoskeletal actin dynamics, lamellipodia formation, and migration (Figure 1B); dissociation of the FAT1 intracellular domain from FAT1 extracellular interactions could hinder directional migration. Whether endogenous forms of the FAT1 intracellular domain can act as physiologic negative regulators of migration is not known.

In addition, FAT1, through its intracellular domain, physically interacts with Atrophin proteins, including Atrophin-1 and Atrophin-2 (also known as arginine-glutamic acid dipeptide repeats, RERE), and colocalizes with these proteins in the perinuclear region, in the nucleus, at cell–cell junctions, and at leading edges in VSMCs [48]. Atrophin proteins have been described as transcriptional corepressors that promote histone deacetylation or methylation to inhibit gene transcription [49]. Like FAT1, the expression of both Atrophins increases in the arterial wall after balloon injury as well as in primary cultured VSMCs in response to serum, PDGF-BB, angiotensin II, basic FGF, and interleukin-1β, known inducers of cell migration [48]. VSMCs express two distinct transcripts for Atrophin-2, which are referred to as short and long isoforms; the chemotactic factors mentioned above induce the short isoform of Atrophin-2 more strongly than the long isoform in VSMCs [48].

**Figure 2:**
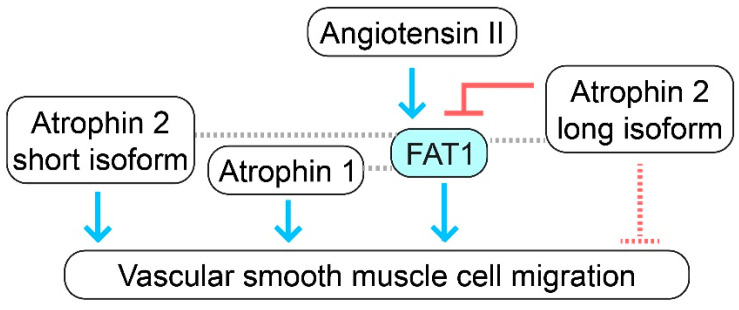
Interactions of FAT1 with Atrophins in vascular smooth muscle migration. FAT1, Atrophin-1, and the short isoform of Atrophin-2 (also known as arginine-glutamic acid dipeptide repeats, RERE) activate VSMC migration. On the contrary, the long isoform of Atrophin-2 inhibits VSMC motility. FAT1 and Atrophin-1 interact physically in VSMCs, and a double knockdown of FAT1 and Atrophin-1 has an additive effect in terms of inhibiting VSMC migration compared to single knockdowns. FAT1 also physically interacts with Atrophin-2; interestingly, like FAT1 knockdown, a simultaneous knockdown of FAT1 and the long isoform of Atrophin-2 decreases VSMC migration, suggesting that the long isoform of Atrophin-2 may serve as a negative upstream regulator of FAT1 in terms of VSMC migration. Angiotensin II acts as a positive upstream regulator of FAT1. Blue arrows indicate stimulatory functional interactions, red symbols indicate inhibitory functional interactions, the dotted red lines indicate an inhibitory function that cannot be ruled out but has not been demonstrated, and dotted gray lines indicate physical interactions.

Interestingly, Atrophin-1 knockdown, like FAT1 loss, impairs VSMC migration, while Atrophin-1 overexpression promotes this cellular activity [48]. A combined knockdown of FAT1 and Atrophin-1 shows an additive effect in inhibiting VSMC motility [48]. On the other hand, the simultaneous knockdown of both Atrophin-2 isoforms does not affect VSMC migration; in contrast, the specific knockdown of the long isoform of Atrophin-2 increases VSMC motility [48]. Notably, the simultaneous knockdown of the long Atrophin-2 isoform and FAT1 decreases VSMC migration, indicating that FAT1 is required for the effect of this long isoform on migration [48]. Altogether, these genetic studies suggest that Atrophin-1 and the short isoform of Atrophin-2, like FAT1, promote VSMC migration; whereas the long isoform of Atrophin-2 inhibits this cellular activity, likely working as an upstream inhibitor of FAT1 (Figure 2). As Atrophin-2 has been described to function as a transcriptional corepressor [49], it is reasonable to suggest that the long isoform of Atrophin-2 may be limiting the expression of gene products that promote VSMC migration, including FAT1. The molecular mechanisms downstream of the interaction of FAT1 with Atrophins in VSMCs are, nevertheless, not fully understood.

On the other hand, angiotensin II may serve as an upstream activator of FAT1 to induce VSMC migration. Angiotensin II increases FAT1 expression and VSMC motility; interestingly, FAT1 knockdown suppresses angiotensin II-induced VSMC migration, indicating that FAT1 is required for this effect of angiotensin II [50]. Mechanistically, angiotensin II-induced FAT1 expression and enrichment at the plasma membrane in VSMCs are abrogated by treatment with valsartan, an angiotensin II receptor 1 blocker, or with apocynin, an inhibitor of NADPH oxidase [50]. In addition, the expression and activity of NADPH oxidase 1 (NOX1) as well as reactive oxygen species generation increase in VSMCs upon angiotensin II treatment; notably, the knockdown of NOX1 suppresses angiotensin II-induced VSMC migration [50]. These studies support the idea that angiotensin II activates angiotensin II receptor 1, which in turn activates NOX1 and ROS generation. Subsequently, these effects lead to increased FAT1 expression at the plasma membrane and enhanced VSMC migration (Figure 1B). The connection between ROS and FAT1 may be mediated by ROS-dependent activation of ERK1/2, as PD98059, an ERK1/2 inhibitor, abrogated angiotensin II-induced FAT1 expression [50].

Altogether, FAT1 expression is stimulated in VSMCs by factors known to promote cell migration and in the arterial wall by conditions characterized by enhanced levels of VSMC migration such as in vascular injury or disease. Full-length FAT1 promotes the migratory activity of VSMCs, and this effect may be mediated downstream in part by the interaction with Ena/VASP proteins, which can connect FAT1 with actin dynamics (Figure 1B). On the other hand, angiotensin II, through NOX1, acts as a positive upstream regulator of FAT1-induced VSMC migration, while the long form of Atrophin-2 may act as a negative upstream regulator (Figure 1B and Figure 2).

## 3. Discussion and Concluding Remarks

Known chemotactic factors (e.g., PDGF-BB, basic FGF, and angiotensin II) induce the expression of the atypical cadherin FAT1 in VSMCs, while injury and disease upregulate FAT1 expression in the arterial wall [9,11]. FAT1, in turn, promotes the directional migration of VSMCs, at least in part by interacting through its intracellular domain with proteins involved in actin polymerization [9]. FAT1-related mechanisms in VSMC migration are nevertheless underexplored at the molecular level. Better understanding of FAT1-mediated VSMC motility may serve as a basis of innovative strategies to modulate this cellular activity and reduce the burden of cardiovascular disease; for example, the disruption of FAT1–Atrophin interactions could potentially limit VSMC orientation and migration and thereby control neointimal expansion.

As noted above, FAT1 promotes the migration of several cell types (skeletal muscle and astrocyte progenitors, fibroblasts, epithelial cells, neurons, podocytes, and renal tubular cells) via cell-autonomous mechanisms; our findings suggest that a similar mechanism holds in VSMCs. On the other hand, FAT1 can either enhance or inhibit the migration of cancer cells depending on the tumor type or context; the molecular basis of this differential effect is not known. It may be that that the released intracellular domain of FAT1, which may translocate to the nucleus or mitochondria, has effects on migration that are opposite to those of full-length FAT1, which is present at the plasma membrane. Another possibility is that the expression of different FAT1 isoforms with distinct subcellular distribution including different regions of the plasma membrane—i.e., leading edge vs. cell–cell contacts—underlies the variable effects on migration observed in different cancer cell types. Explaining how these distinct FAT1-mediated effects on cell motility occur may lead us to a broader understanding of cell migration. A further consideration follows from the observation that the global loss of FAT1 causes CNS, renal, and ocular abnormalities and perinatal lethality in mice [51], which aligns with a role of FAT1 in neuronal function, angiogenesis, glomerular nephropathy, and astrocyte physiology, as noted above. Our studies utilizing VSMC-targeted conditional inactivation of the Fat1 gene show apparently normal development but abnormal post-injury vascular remodeling [11], suggesting that redundancies controlling VSMC activities that are present in development are not functional in the fully formed vasculature.

Our understanding of the functions of the processed intracellular domain of FAT1 in VSMC biology is incipient. Although the FAT1 intracellular domain goes to mitochondria and limits cellular respiration and VSMC proliferation [11], a role in VSMC migration is not yet proven. Intriguingly, engineered overexpression of the FAT1 intracellular domain inhibits VSMC migration [9]; however, no evidence of a related endogenous mechanism has been provided. Further studies of the released FAT1 intracellular domain and potential expression of FAT1 isoforms in VSMC biology are necessary to address these points.

## Data Availability

No new data were created or analyzed in this study, so data sharing is not applicable to this article.
